# Deep learning for image-based large-flowered chrysanthemum cultivar recognition

**DOI:** 10.1186/s13007-019-0532-7

**Published:** 2019-12-04

**Authors:** Zhilan Liu, Jue Wang, Ye Tian, Silan Dai

**Affiliations:** 1 College of Landscape Architecture, Beijing Forestry University, Beijing, 100083 China; 2 College of Technology, Beijing Forestry University, Beijing, 100083 China

**Keywords:** *Chrysanthemum* × *morifolium* Ramat., Deep learning, Image recognition, Grad-CAM

## Abstract

**Background:**

Cultivar recognition is a basic work in flower production, research, and commercial application. Chinese large-flowered chrysanthemum (*Chrysanthemum* × *morifolium* Ramat.) is miraculous because of its high ornamental value and rich cultural deposits. However, the complicated capitulum structure, various floret types and numerous cultivars hinder chrysanthemum cultivar recognition. Here, we explore how deep learning method can be applied to chrysanthemum cultivar recognition.

**Results:**

We propose deep learning models with two networks VGG16 and ResNet50 to recognize large-flowered chrysanthemum. Dataset A comprising 14,000 images for 103 cultivars, and dataset B comprising 197 images from different years were collected. Dataset A was used to train the networks and determine the calibration accuracy (Top-5 rate of above 98%), and dataset B was used to evaluate the model generalization performance (Top-5 rate of above 78%). Moreover, gradient-weighted class activation mapping (Grad-CAM) visualization and feature clustering analysis were used to explore how the deep learning model recognizes chrysanthemum cultivars.

**Conclusion:**

Deep learning method applied to cultivar recognition is a breakthrough in horticultural science with the advantages of strong recognition performance and high recognition speed. Inflorescence edge areas, disc floret areas, inflorescence colour and inflorescence shape may well be the key factors in model decision-making process, which are also critical in human decision-making.

## Background

**Fig. 1:**
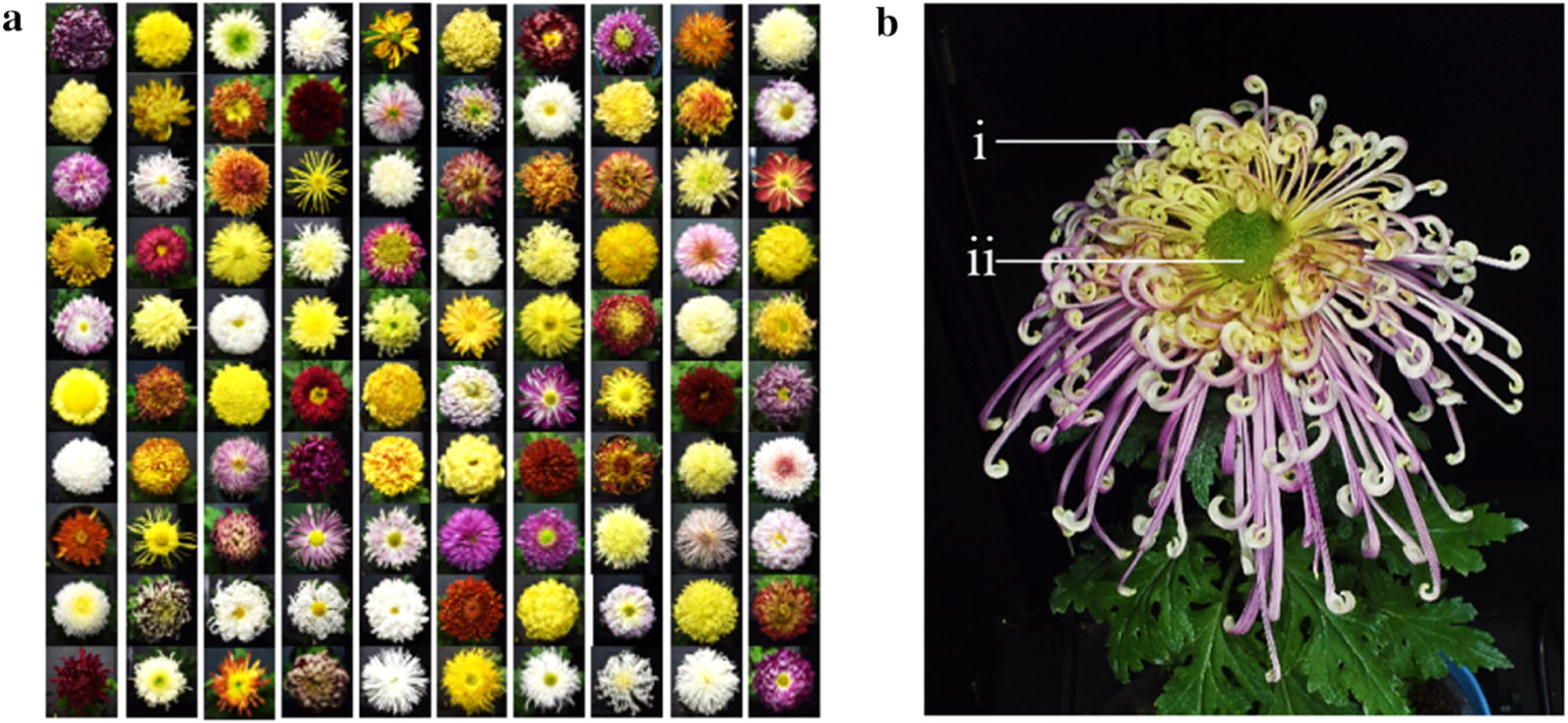
Complexity of chrysanthemum recognition. **a** Thousands of large-flowered chrysanthemum cultivars. **b** The chrysanthemum capitulum structure: (i) ray floret and (ii) disc floret

*Chrysanthemum* × *morifolium* Ramat., which originated in China, has high ornamental and commercial value in the floriculture industry around the world [1–3], and its major production areas cover China, Japan, the Netherlands and South Korea [4]. The Chinese large-flowered chrysanthemum cultivar group is one of the largest cultivar groups of chrysanthemum [5]. The growing number of new chrysanthemum cultivars bred around the world has made it harder to recognize, even if professional researchers may confuse chrysanthemum cultivar, which caused severe loopholes in the management and protection of chrysanthemum resources. Many difficulties have been encountered in the cultivar recognition of large-flowered chrysanthemums, due to the large number of cultivars [2, 5] (Fig. 1a), the complex capitulum structure, the various floret types [6–8] (Fig. 1b), and the highly heterozygous genetic background [9, 10]. Thus, it is extremely challenging to recognize chrysanthemums accurately and rapidly.

In previous studies, the traditional morphological method, the comprehensive mathematical statistics method and molecular markers were used to solve the large-flowered chrysanthemum recognition problem. The traditional method utilized human decision-making based on various morphological characteristics, e.g., flower diameter, colour, florescence and flower type [11–13]. Numerical taxonomy and multivariate statistical analysis made the recognition process more objective and quantitative [3, 14–16], but required labourious and time-consuming manual measurements. Molecular markers have the potential to recognize cultivars with similar morphological features [17], but required laboratory testing over long periods of at least half a day. At present, the rapid recognition of large-flowered chrysanthemums is difficult to achieve.

Image-based deep learning methods have been increasingly applied in the plant recognition field [18, 19] with the high-speed development of machine learning, which used machine self-learning from massive image data to identify the key features [20]. Compared with previous manual measurement methods, image capture could quickly transform plant morphological information to two-dimensional image information, thus it substantially simplified the process of plant phenotypic data collection [21]. Deep convolutional neural network (DCNN) has been used to identify thousands of plant species. Two deep learning architectures, namely, GoogLeNet and AlexNet, and 8189 images were used to recognize 102 flower species [22]. Flower recognition applications, such as Flowers Partner [23], Flower Recognition [24] and XingSe [25], could recognize more than 4000 plant species based on the deep learning framework. Increases in data availability, along with advances in DCNNs, have made the related approaches more accurate, faster, and cheaper; hence, these approaches have the potential to significantly contribute to solving the problem of flower recognition. Well-trained automated plant recognition systems are now considered to be comparable to human experts in labelling plant on different images [26].

In this paper, we trained a DCNN classifier and used it to recognize large-flowered chrysanthemum cultivars. To the best of our knowledge, the perspective affects the recognition results. A balanced dataset, namely, dataset A, was constructed from 14,000 images of 103 cultivars. The images in dataset A were captured by using an automatic image acquisition device to photograph from predefined perspectives. Each image was reviewed via manual examination to ensure the accuracy of cultivar recognition. Dataset B was constructed from 197 images that were captured in previous years and was used to evaluate the model generalization performance, and the feature distribution of cultivars was observed via T-distributed stochastic neighbour embedding (T-SNE). In addition, the gradient-weighted class activation mapping (Grad-CAM) method and feature clustering were used to interpret the deep learning model’s decision-making process from the human perspective. In the study, the main objective was to establish an image-based DCNN classifier for chrysanthemum recognition that would provide high reference value for flower cultivar recognition and plant classification research.

## Methods

### Image dataset A

#### Cultivar selection and plantation

**Fig. 2:**
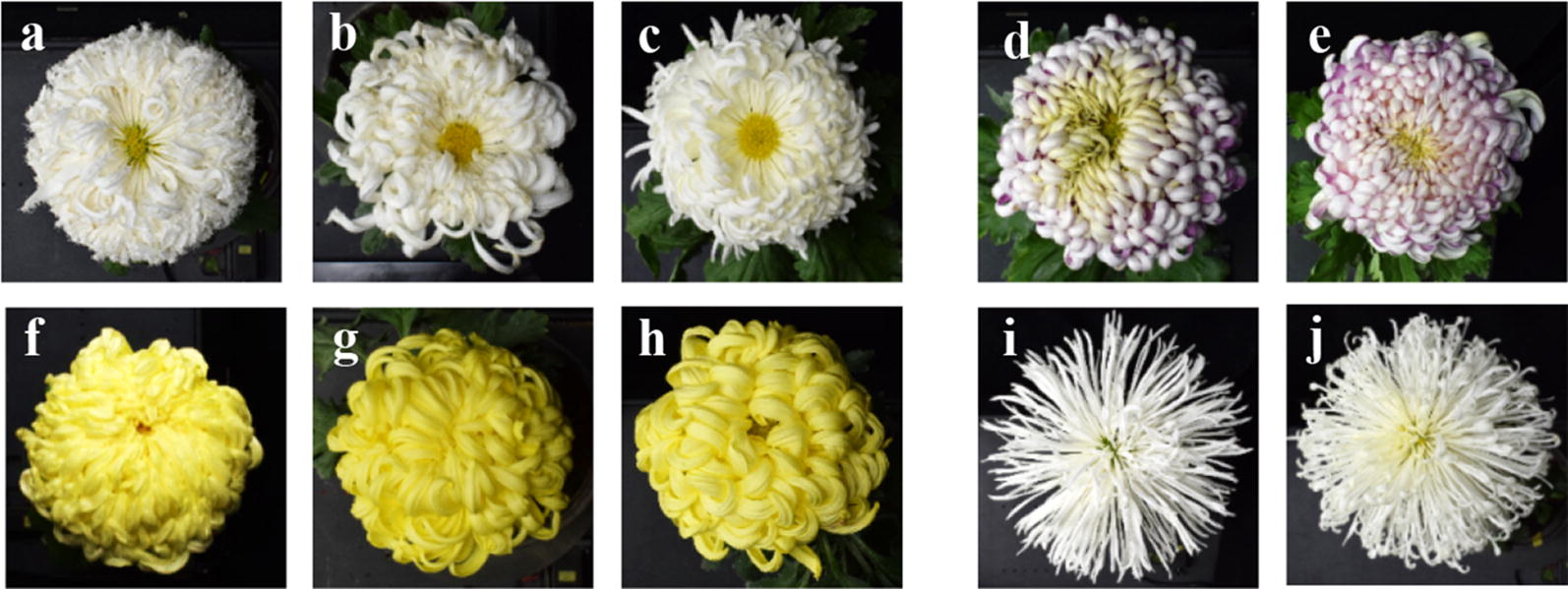
Similar cultivars in terms of morphology. **a**–**c**, **b**–**d**, **e**–**g**, and **h**–**j** are similar cultivars. The cultivar names are **a** ‘Baifenshizi’, **b** ‘Qiongdaoshanyou’, **c** ‘Zilangfengguang’, **d** ‘Zilongwoxue’, **e** ‘Tangyuqiushi’, f ‘Jinfomian’, **g** ‘Jinshitou’, **h** ‘Tangyujinqiu’, **i** ‘Yulingguan’, and **j** ‘Baisongzhen’

The experimental material is traditional Chinese large-flowered chrysanthemum cultivar group. 103 cultivars were selected (see Additional file 1: Fig. S1), and some of them were similar in terms of morphology (Fig. 2). The cultivar naming standard of the Chinese Chrysanthemum Book was utilized [5].

Plantation work was carried out in the nursery of Beijing Forestry University (Beijing, China) from April 2017 to September 2017. Our cultivation was single-flower cultivation with 10 samples per cultivar. The process included cutting, pot changing, and colonization [8]. Water, fertilizer, insects, and disease control measures were supplied during this period.

#### Image acquisition

An automatic image acquisition device, which was designed by our researchers and the GreenPheno Company (Wuhan, China), was used to acquire images (80–200 images per cultivar) from October 2017 to December 2017. The flowerpot was placed on a horizontal rotation platform (Additional file 1: Fig. S2), and the rotation angle could be controlled by a computer. When the device rotation stopped, the flowerpot was automatically captured by three cameras from the top, oblique and side views. Because the cultivar height ranged from 0.5 to 1.5 m, we designed a special mode to ensure that the image was focused. First, the flower height was determined by the side-view camera. Then, top-view and oblique-view cameras automatically moved up or down to maintain a specified focusing distance from the top of the flower. Additional details about this device are provided in Additional file 1: Figs. S2 and S3.

After image acquisition, all the images were manually annotated by two researchers over a month, and locally unfocused images were cleaned accordingly.

#### Dataset construction

**Fig. 3:**
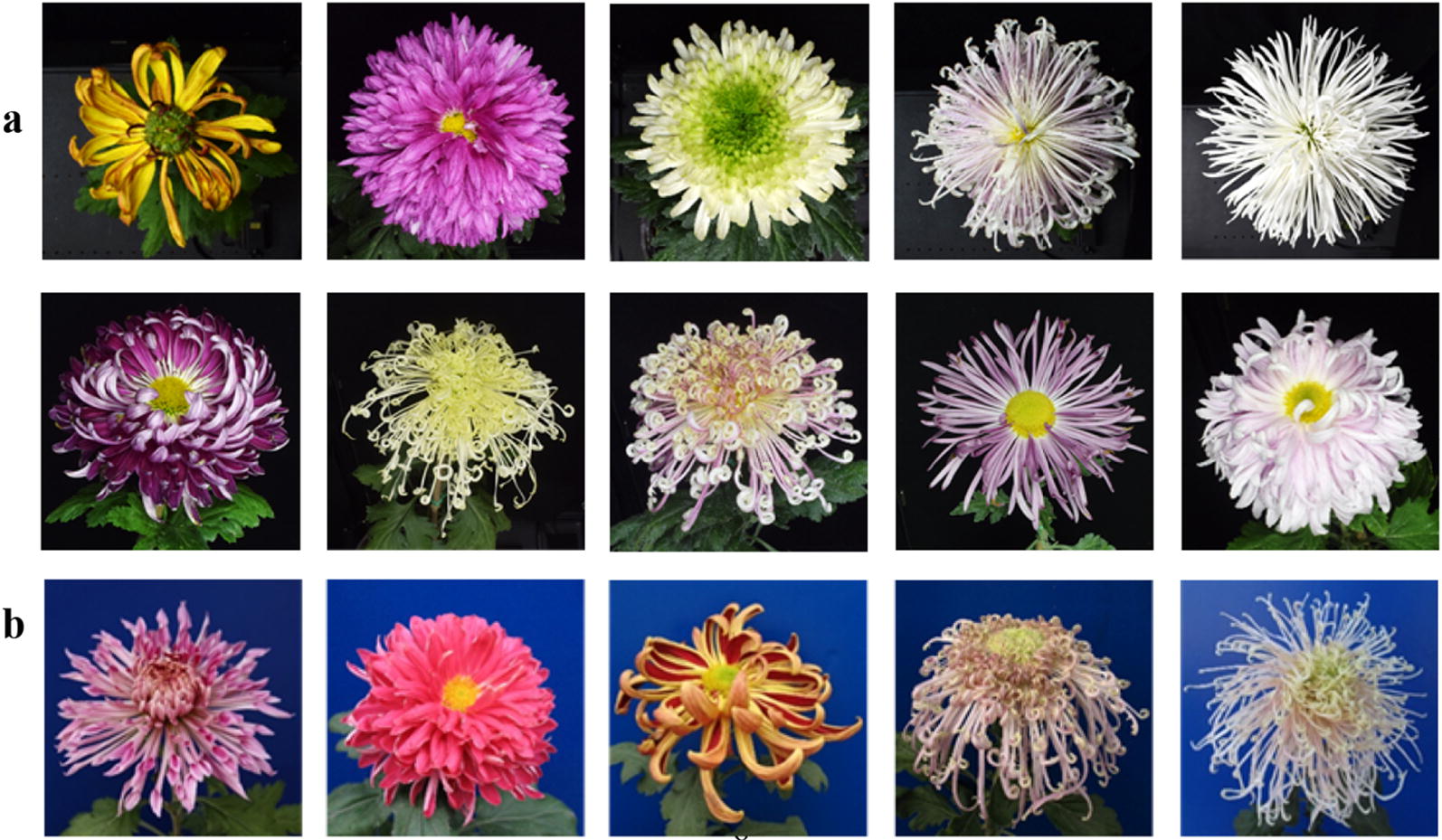
Sample images from dataset A (**a**) and B (**b**)

Chrysanthemum dataset A (Fig. 3a) contains 14,000 images (PNG format) of 103 cultivars of Chinese large-flowered chrysanthemum that were captured in 2017. Preserving the percentage of samples for each class, we randomly divided all images into subsets (training, validation and testing).

### Image dataset B

Chrysanthemum dataset B contains 197 images (2–3 images per cultivar) of the same cultivars as in dataset A (Fig. 3b). The images were captured by our group with a digital camera (Canon EOS 750D) in 2008–2010 and in 2016. Compared to the cultivars in 2017, the same cultivars in those years had different cultivating conditions and climatic environment, which led to subtle changes in dataset B. In addition, the images that were captured via manual shooting have higher flexibility compared to machine shooting. To measure the model generalization performance, 197 images were imported into the established classifier.

### DCNN approach

#### Devices

The DCNN models were trained on the Ubuntu 16.04 system on an NVIDIA TitanV GPU (Intel Xeon Gold 5120) hardware platform using the Deep Learning GPU Training System (DIGITS) software, which was developed by NVIDIA.

#### Framework

Two pre-trained networks, namely, VGG16 and ResNet50 [27, 28], which were the models in the ImageNet Large Scale Visual Recognition Competition (ILSVRC) in 2014 and 2015, were selected for the detection of a suitable model for chrysanthemum recognition.

**Fig. 4:**
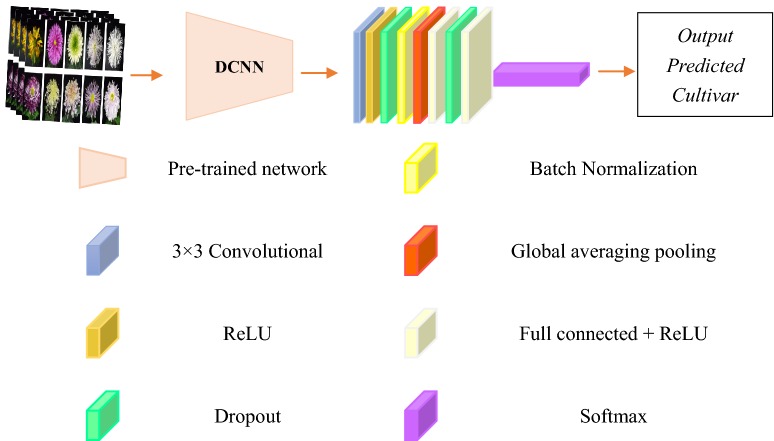
DCNN framework. Batch normalization was used to improve the performance and stability of DCNN. Dropout prevented DCNN from overfitting. Global average pooling can adapt to any input image size

In contrast to many common classification tasks of classifying images of various species, such as the ImageNet task, our objective was to classify the images of one species into various cultivars. Hence, the model must extract the fine features of the callosity patterns regardless of the image perspective. Dataset A is a small dataset with only 14,000 images. A common and highly effective approach for deep learning on small image datasets is to use a pre-trained network or transfer learning. A pre-trained network is a saved network that was previously trained on a large dataset, typically on a large-scale image-classification task. The spatial hierarchy of features that was learned by the pre-trained network is used to effectively serve as a generic model of the visual world; hence, its features can be used for many computer vision problems, even completely different classification tasks than the tasks for which it was trained [29]. There are two ways to use the pre-trained network: feature extraction and fine-tuning. Feature extraction was adopted in our DCNN. DCNN is composed of feature extractors and classifiers. The feature extractors that are learned by the pre-trained network have been proven effective in many computer vision problems. Therefore, a classification model can be built on the basis of the pre-trained network. In Fig. 4, VGG16 or ResNet50 acts as a feature extractor. The classifier is comprised of two fully connected layers (each includes 4096 hide units), a global averaging pooling layer and a dropout layer (0.25). The activation function is ReLU.

We used VGG16 and ResNet50 as the base architectures of the network. Although VGG16 is an older model that is far from the current state of the art and is heavier than many recent models, its architecture is simple, and it is easy to understand how the network obtains its final classification decision for a specified image. ResNet50 is famous for obtaining remarkable results on various image classification and object detection tasks. In some respects, ResNet50 is a state-of-the-art method.

#### Training

DCNN requires a constant input dimensionality. Hence, the flower objects were cropped out from the original images (6000 × 4000 pixel), and the processed images (2000 × 2000 pixel) were down-sampled to a fixed resolution. We trained our DCNN models on the raw RGB values of the pixels. To obtain fixed-size (224 × 224 pixel) DCNN input images, the rescaled-size (256 × 256 pixel) training images were randomly cropped (one crop per image per iteration). To further augment the training set, the crops underwent random transformations, including rotation, translation, shearing, and random RGB colour shifting.

Via the transfer learning approach, a classification model could be built on the basis of the pre-trained network. Two pre-trained networks (VGG16 and ResNet50) were used to compare the classification results. Training used a batch size of 64 with a learning rate of 0.01 and was terminated after 20 epochs. The learning rate adjustment method was the STEP method. As an optimizer for our training algorithms, stochastic gradient descent (SGD) was used. After the training set image was completed, it was verified. The set was tested once in batches.

#### Evaluation

**Fig. 5:**
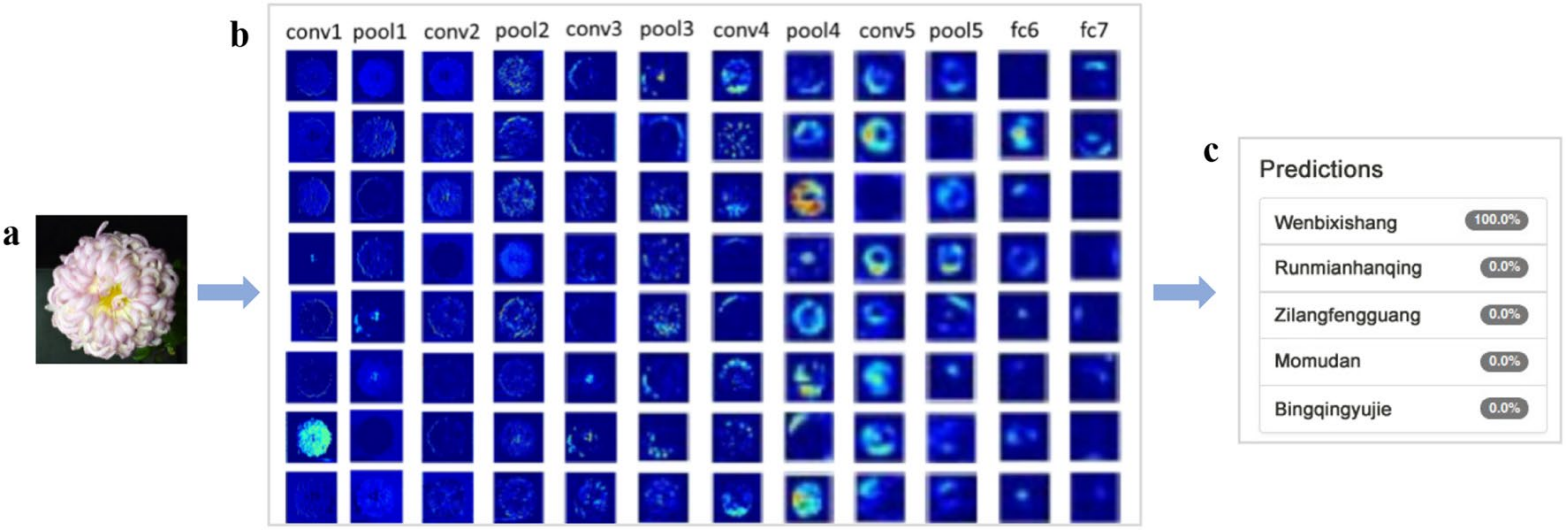
VGG16 model recognition. **a** An input image. **b** The feature extraction visualization results of each convolutional (conv), pooling and fully connected (fc) layer in VGG16 after transfer learning. **c** The Top-k results

As our dataset was completely balanced, we could simply calculate the Top-1 and Top-5 accuracies for each cultivar as the averages across all images of the test set. Top accuracies have been widely used to evaluate DCNN models in computer vision and image classification; e.g., nearly all papers that present DCNN models that were evaluated on the ImageNet dataset presented their results in terms of both the Top-1 and Top-5 accuracies. Therefore, we applied the Top accuracies to our DCNN classifier that was trained on the Chrysanthemum dataset. The Top-1 accuracy is the percentage of predictions for which the top prediction matches the ground-truth label; to calculate this value, the total number of correct predictions is divided by the number of data points in the dataset. When working with Chrysanthemum dataset, which included many class labels with similar characteristics, we could examine the Top-5 accuracy as an extension of the Top-1 accuracy to evaluate the performance of our network (Fig. 5).

### T-SNE

T-SNE could be used to observe the distribution of the chrysanthemum dataset. T-SNE is a method for representing the spatial distribution of features [30], and it projects high-dimensional data into two-dimensional or three-dimensional visualizations to observe the depth features of the cultivars in a spatial distribution, which is used to visually assess the model classification performance. The main strategy is to use the joint probability distribution *p*_*ij*_ with symmetry to represent the distances between the sample points in the high-dimensional space [30]. In this paper, we used the T-SNE algorithm to observe the distribution of the high-dimensional features of each image. We extracted the 4096-dimensional features from the 7th layer (fc7) of the chrysanthemum recognition model (VGG16 as a pre-trained network) for each image in the dataset training set (11,200 images), and we visualized the high-dimensional features in two-dimensional space. The perplexity was 50 and n_iter was 2000.

### Grad-CAM visualization

DCNNs are often described as ‘black-boxes’; they learn feature representations that are difficult to interpret in a human-readable form. However, the behaviours of DCNNs may be interpreted via Grad-CAM visualization [31]. This approach has been applied to image analysis of soybean plant disease leaves [32]. The main strategy of this approach was to extract the information of the last feature map of the convolutional network (VGG16 as a pre-trained network) to weight the corresponding gradient to produce a location map for displaying key recognition areas in the image [31]. Via Grad-CAM visualization, we could determine which parts of a chrysanthemum image were important when it was identified as belonging to a specified class.

### Feature clustering analysis

A 4096-dimensional feature was extracted in the 7th activation layer of the model (VGG16 as a pre-trained network) from each of the 103 cultivar images (5 images per cultivar), and the features of 5 images were averaged for each cultivar. The features could reflect the understanding of model regarding each cultivar image. Hierarchical clustering analysis was conducted on the features of 103 cultivar images using MATLAB 2014a (MathWorks, MA, USA). The calculated distance was set as the cosine distance, the measurement method was ward and the clustering method was the shortest-distance method.

## Results

### Model accuracy performance

Top-k accuracy rate indicators on dataset A were used to the model evaluation. Table 1 lists the calibration accuracies of the VGG16 and ResNet50 networks. The results demonstrate that the recognition accuracies are sufficiently high.

**Table 1:** Top-1 and Top-5 calibration accuracies for VGG16 and ResNet50 on dataset A

Pre-trained network	Top-1 (%)	Top-5 (%)
VGG16	89.43	98.59
ResNet50	95.39	99.51

### Model generalization performance

**Fig. 6:**
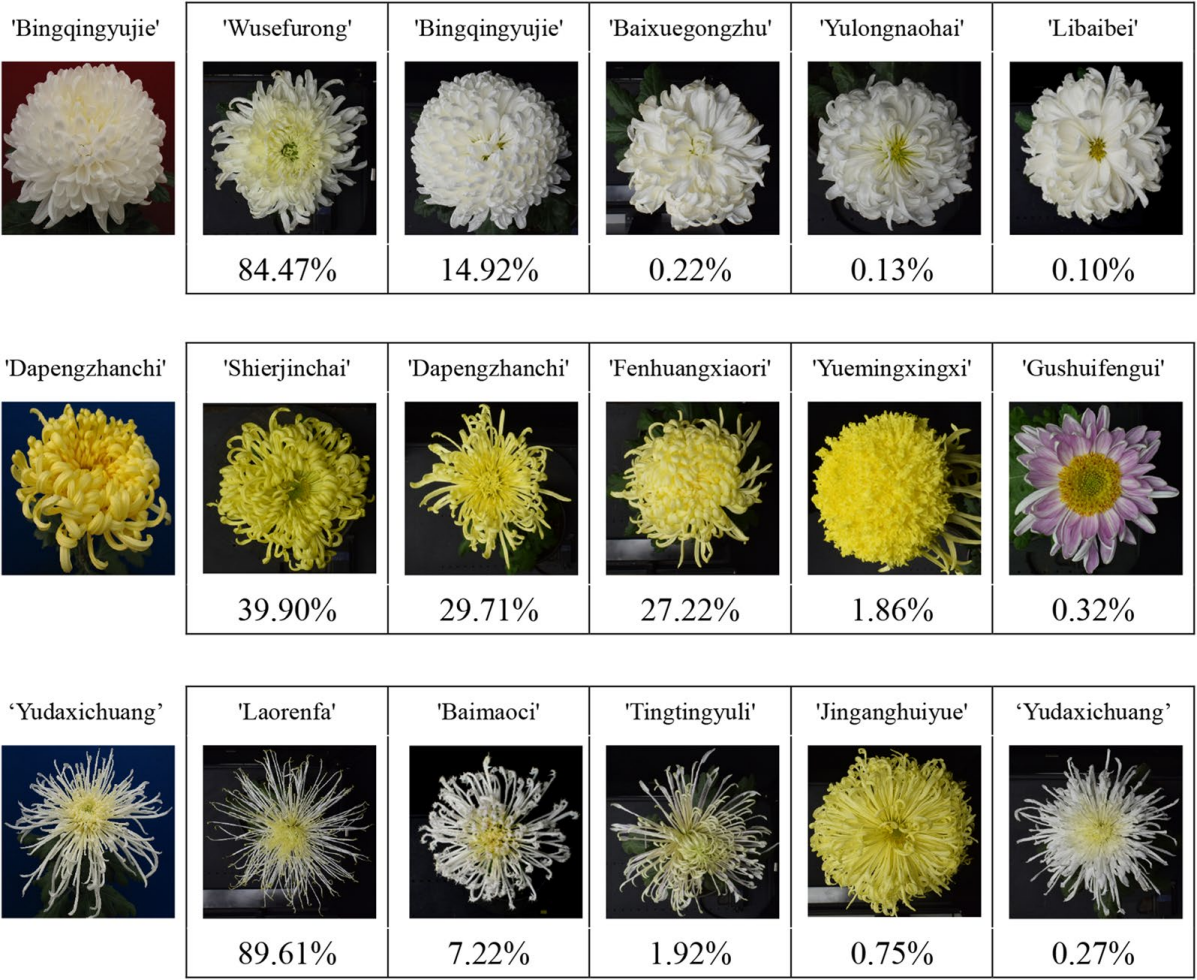
Top-5 recognition results. On the top of each image, the cultivar name is specified, such as 'Bingjingyujie'; on the bottom, the recognition accuracy is specified

The generalization performance is the recognition performance of the DCNN model on new images. Dataset B, which differs substantially from dataset A, was used to measure the model generalization performance. According to Table 2, the two network structures achieved high recognition accuracies. ResNet50 achieved an accuracy of 69.86%. Figure 6 presented the cases that correspond to the Top-5 recognition results. Many cultivars in the Top-5 prediction list were highly similar morphology, especially in terms of flower colour.

**Table 2:** Top-1 and Top-5 accuracies for VGG16 and ResNet50 on dataset B

Pre-trained network	Top-1 (%)	Top-5 (%)	Analysis time per image (ms)
VGG16	51.93	78.21	10
ResNet50	69.86	88.19	15

### Feature distribution

**Fig. 7:**
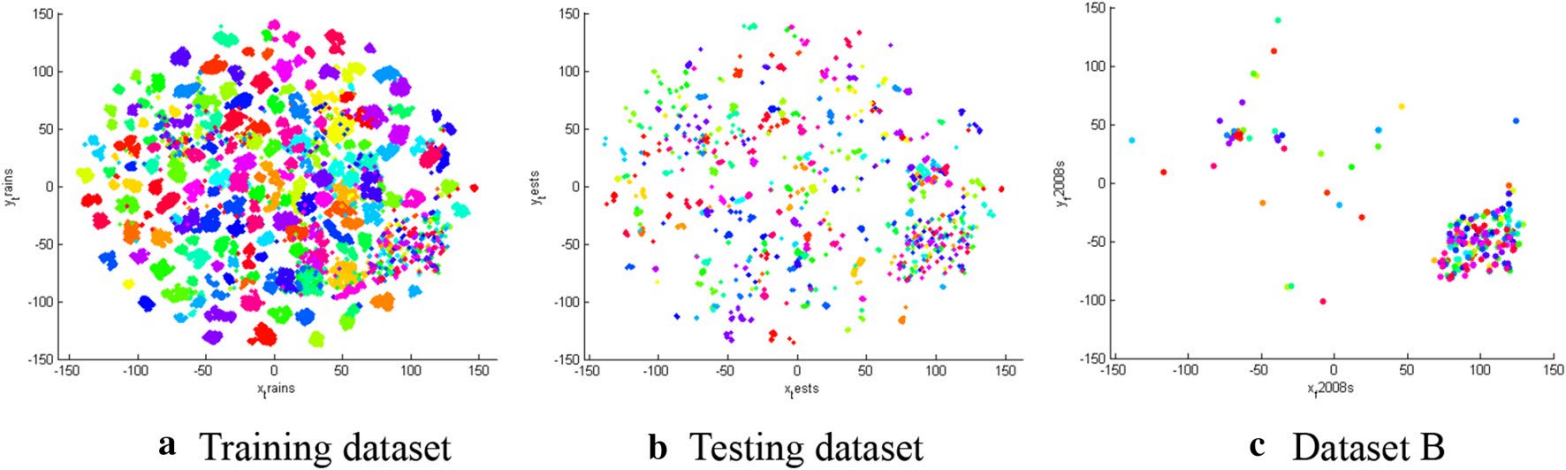
Feature distribution maps. Each point represents a high-dimensional feature of an image and each colour represents a label of cultivars

The distribution of the features were extracted from the various cultivar images by the model that was based on the ResNet50 network. The features of the images of the same cultivar had strong aggregation characteristics (Fig. 7); hence, the features could accurately describe the various chrysanthemum cultivars.

### Model decision-making process analysis

**Fig. 8:**
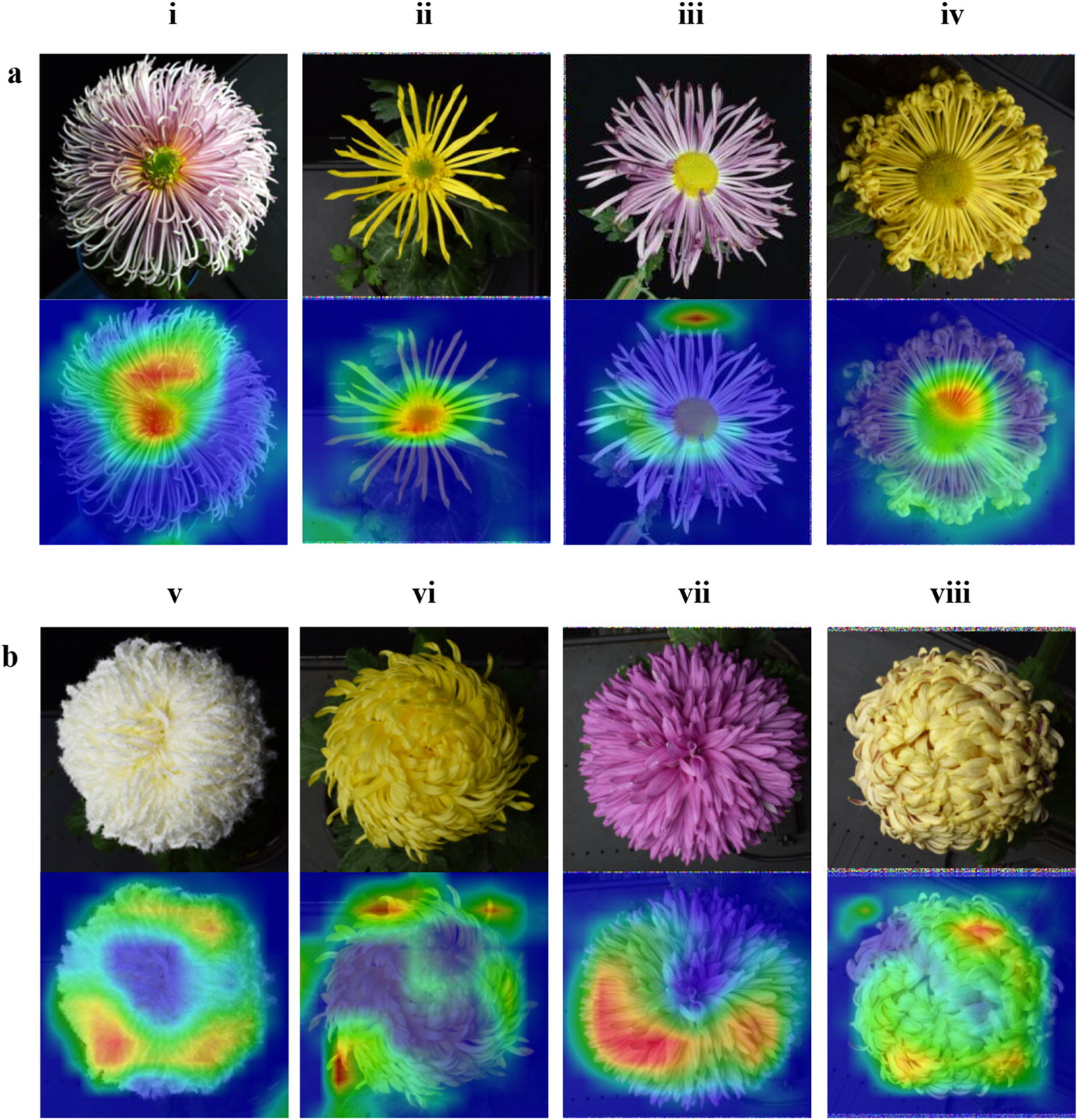
Heat maps of eight large-flowered chrysanthemum images. **a** Cultivars whose centre disc florets are visible and **b** cultivars whose centre disc florets are invisible. Eight cultivars of chrysanthemum are shown in i–viii. In each heat map, the warmer the pixel's colour, the more attention the model pays to it. The red areas represent the most critical recognition areas of the model

According to the heat maps that were generated by the Grad-CAM method (Fig. 8), the model paid substantial attention to inflorescence edge areas and disc floret areas, and it paid little attention to the leaves and the black background. For the cultivars whose centre disc florets were visible (Fig. 8a), the model focused on the centre disc floret areas, followed by the inflorescence edge areas. For the cultivars whose centre disc florets were invisible (Fig. 8b), the model focused on the inflorescence edge areas. In summary, the inflorescence edge areas and disc floret areas were the key recognition positions.

**Fig. 9:**
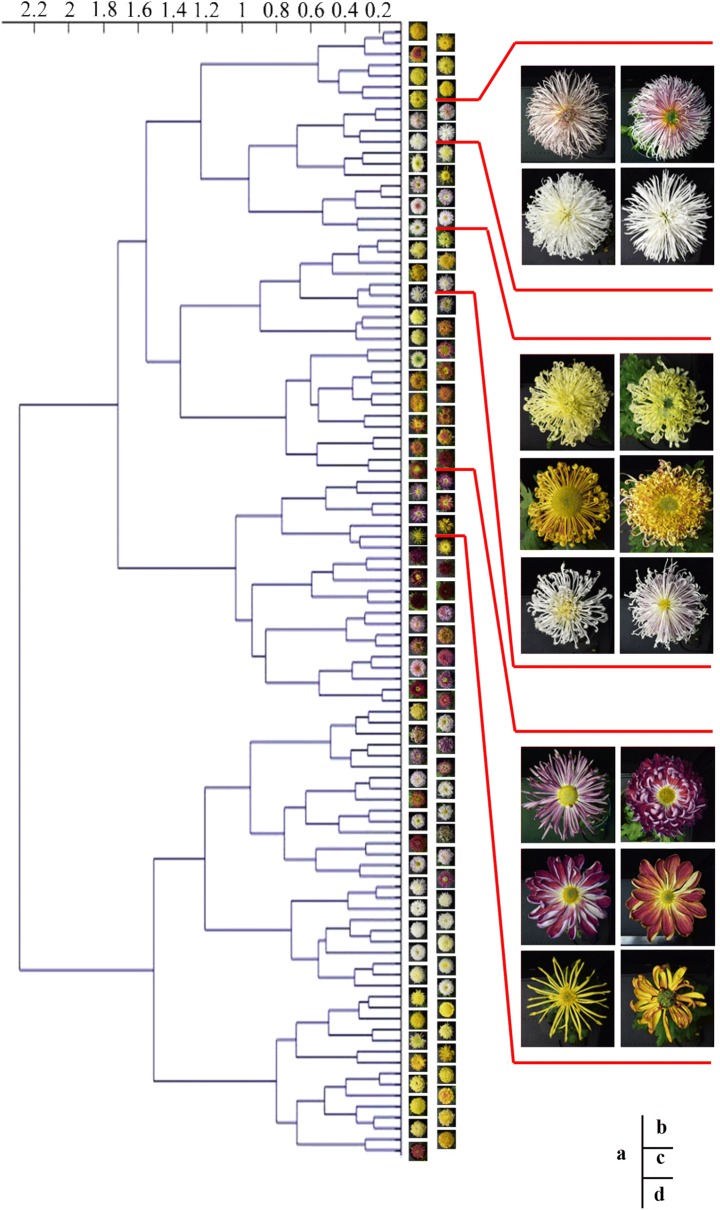
Clustering analysis of features of 103 cultivar images

Clustering analysis was carried out on the 103 cultivar image features, which are presented in a tree diagram (Fig. 9). When the distance was 1.8 to 2.2, 103 cultivar image features were clustered into two categories, which had little readily observable morphology regularity between the corresponding cultivar images (Fig. 9a). When the distance was 1 to 1.2, the cultivars with similar colour were clustered together, especially white and yellow cultivars (Fig. 9a). When the distance was 0.6 to 0.8, the cultivars with similar flower shape were clustered together (Fig. 9b–d). In summary, the inflorescence colour and shape were highly correlated with the features that were extracted by the model.

## Discussion

### Advantages of image-based deep learning method

Plant morphological identification is conducted by the naked eye via qualitative comparison to identify the differences in shape among species. It is difficult to transform species information into data for statistical mathematical analysis [33]. Compared with traditional morphological data collection methods, the image acquisition method has the advantages of high speed, high efficiency and ability to carry large amounts of information, thereby substantially simplifying the process plant phenotypic data collection [21, 34].

Deep learning method is a potential research method for identifying plants [35–38]. Compared to inter-species recognition [39], cultivar recognition with high intra-class variability and small inter-class differences is a more challenging task. Compared to traditional methods, which take at least half a day, the DCNN models took 10 ms (VGG16) and 15 ms (ResNet50) to analyse a single chrysanthemum image (Table 2), rapidly. In addition, the DCNN that is utilized in this paper can recognize cultivars with high similarity in terms of morphology, whereas applied shallow learning methods [40, 41] performed poorly on similar chrysanthemum cultivars. In addition to birds [42], cats and dogs [43] and other classification tasks, this paper demonstrates that the deep learning method also performs well in the classification of chrysanthemum cultivars.

### New challenges of chrysanthemum recognition

Although deep neural networks have high recognition accuracy, their lack of decomposability into intuitive and understandable components renders them difficult to interpret [44]. As the large-flowered chrysanthemum phenotype is highly complex, traditional morphological methods must be combined with multiple traits [3, 14, 16]. How does the deep learning model recognize chrysanthemum? We found that the inflorescence centre and edge areas are important recognition areas and that chrysanthemum images with similar colour or flower shape are always clustered in the same class. This paper reports our initial attempt at clustering analysis. Although our paper did not define the key recognition features of large-flowered chrysanthemums, it demonstrated a fruitful exploration process.

**Fig. 10:**
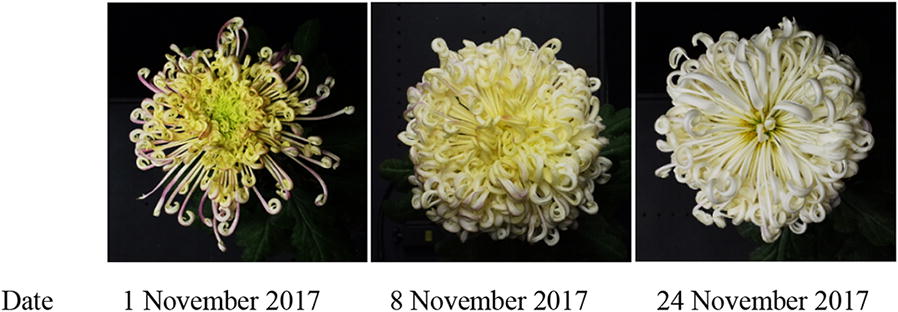
Opening process of a large-flowered chrysanthemum. Note: The flower that is shown is ‘Fengguanxiapei’

Since hardly any professional chrysanthemum dataset was available online, we established a high-definition large-flowered chrysanthemum image database by recording, storing and sorting chrysanthemum images, which has important significance and value for chrysanthemum image research. A large set of image data is the training basis of a deep learning model. Moreover, the plant phenotype may change with the spatial and temporal conditions. By continuously photographing the same chrysanthemum, we found that some cultivars’ phenotypes dramatically changed during their flower opening process, as shown in Fig. 10. Therefore, researchers must collect phenotypic images of the same cultivar in various states, which will pose new challenges in the chrysanthemum recognition task in the future.

## Conclusions

A new method for large-flowered chrysanthemum cultivar recognition is proposed in this paper. The ideal application is that by uploading a single chrysanthemum image to our system, researchers can quickly obtain the Top-5 cultivar information for predicting the cultivar name with the corresponding cultivar images from the system. In addition, newly uploaded images could be reused as input samples for the next iteration, which continuously improves the generalization performance of the model.

## Supplementary information

**Additional file 1: Fig. S1.**Device images of 103 chrysanthemum cultivars (top-view image). **Fig. S2.** Schematic diagram of the automatic chrysanthemum image acquisition device. **Fig. S3.** Automatic chrysanthemum image acquisition device.

## Data Availability

The datasets that were used and analysed during the current study are available from the corresponding author upon reasonable request.

## Abbreviations

DCNN: deep convolutional neural network
ILSVRC: imageNet Large-Scale Visual Recognition Competition
SGD: stochastic gradient descent
T-SNE: t-distributed stochastic neighbour embedding
Grad-CAM: gradient-weighted class activation mapping
