# Supplementary material for: Deep learning for image-based large-flowered chrysanthemum cultivar recognition
Source: Plant Methods. 2019 Dec 4;15:146. doi: 10.1186/s13007-019-0532-7 (PMC6892201; doi:10.1186/s13007-019-0532-7)
Supplement: Supplementary file 1 — Additional file 1: Fig. S1.Device images of 103 chrysanthemum cultivars (top-view image). Fig. S2. Schematic diagram of the automatic chrysanthemum image acquisition device. Fig. S3. Automatic chrysanthemum image acquisition device. [file 13007_2019_532_MOESM1_ESM.docx]

| 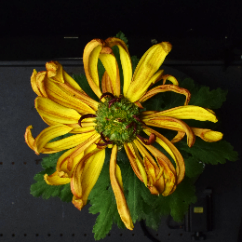  ‘Xuri’ | 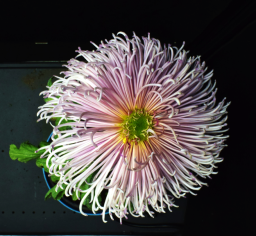 ‘Bainiaochaofeng’ | 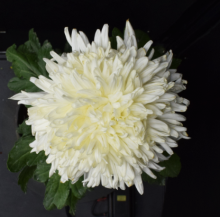 ‘Yulongxishui’ | 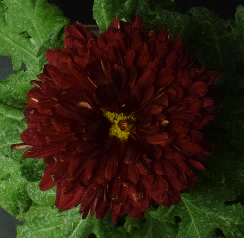 ‘Daidaichaoxia’ |
| --- | --- | --- | --- |
| 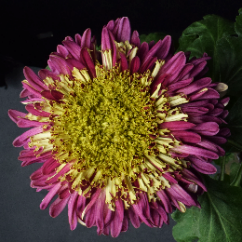 ‘Ziruigong’ | 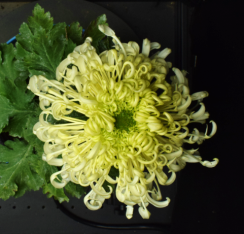 ‘Lvyun’ | 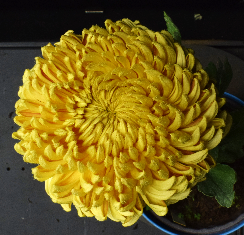 ‘Gushajingang’ | 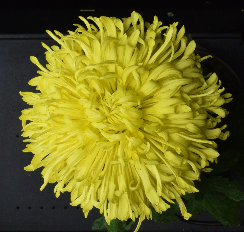 ‘Jinlingguan’ |
| 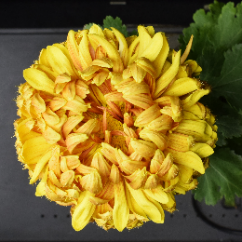 ‘Xishanhongri’ | 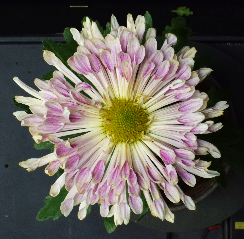 ‘Xiuhuapo’ | 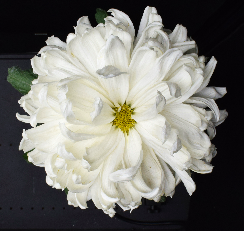 ‘Yulanhe’ | 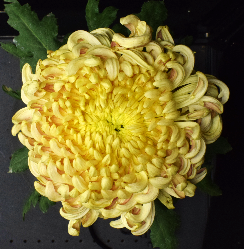 ‘Tiangezhuiyu’ |
| 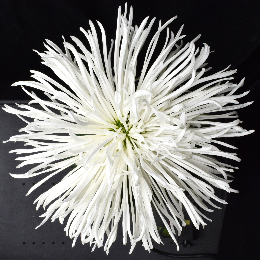 ‘Yulingguan’ | 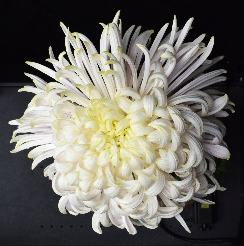 ‘Yulouchun’ | 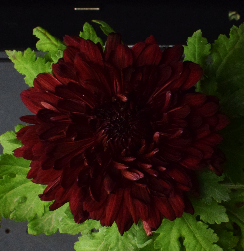 ‘Morong’ | 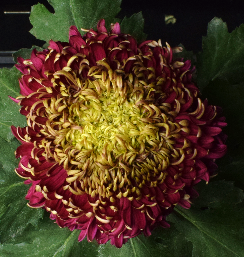 ‘Mokui’ |
| 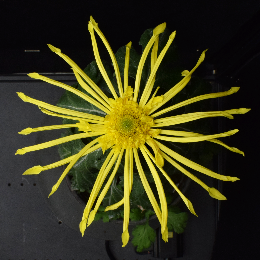 ‘Changongguise’ | 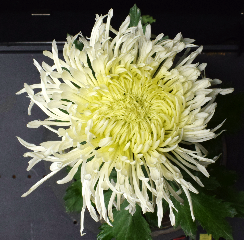 ‘Luhuayueying’ | 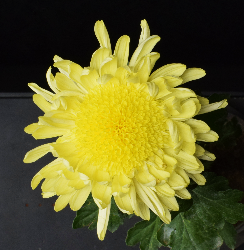 ‘Huanshuijingui’ | 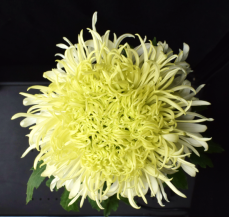 ‘Bailuhengjiang’ |
| 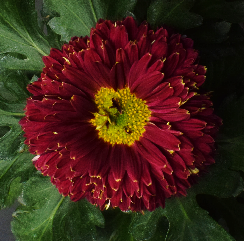 ‘Tianxiayipin’ | 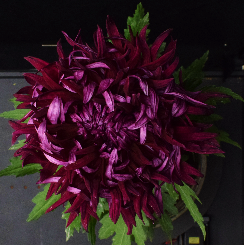 ‘Yongshoumo’ | 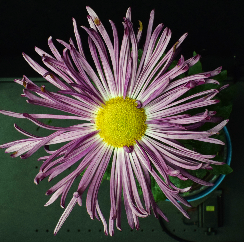 ‘Zixianchuanzhen’ | 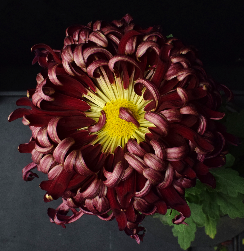 ‘Moxie’ |
| 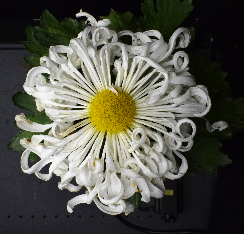 ‘Yuhudie’ | 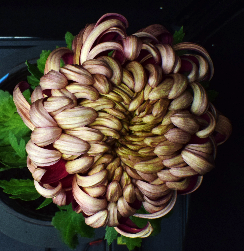 ‘Hubeixieyang’ | 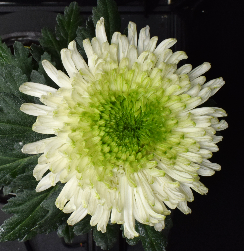 ‘Lvbaoshi’ | 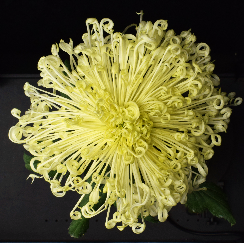 ‘Feixueyingchun’ |
| 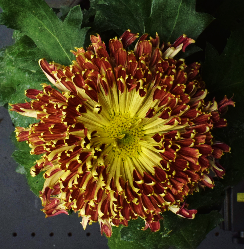 ‘Jinlongxianxuezhua’ | 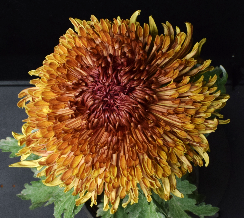 ‘Zhushaguanzhu’ | 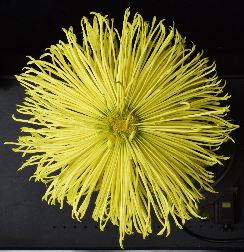 ‘Jinxiaguan’ | 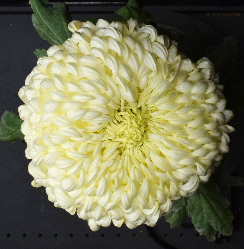 ‘Bingxinzaibao’ |
| 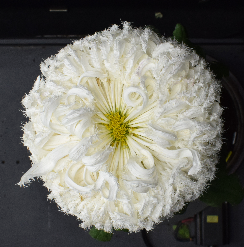 ‘Baifenshizi’ | 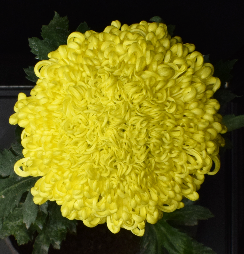 ‘Jinboyongcui’ | 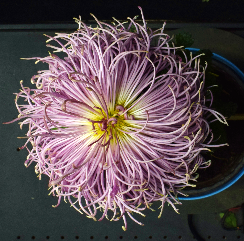 ‘Zhinv’ | 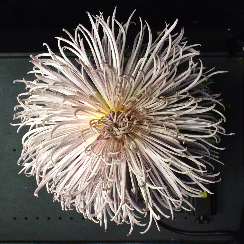 ‘Huihexianzhu’ |
| 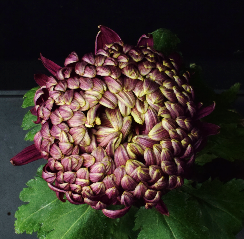 ‘Momudan’ | 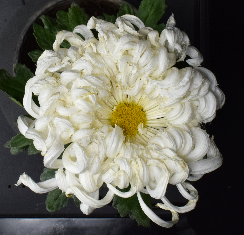 ‘Qiongdaosanyou’ | 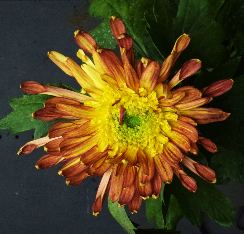 ‘Biyutuogui’ | 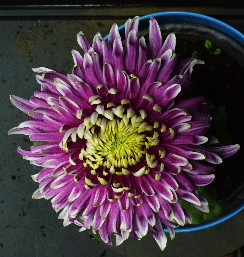 ‘Taohongliulv’ |
| 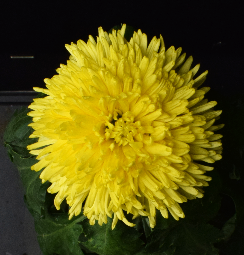 ‘Huangguanqiu’ | 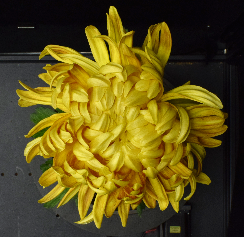 ‘Jinxiuyuanyang’ | 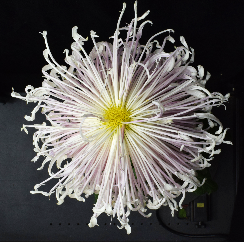 ‘Feizhusanxia’ | 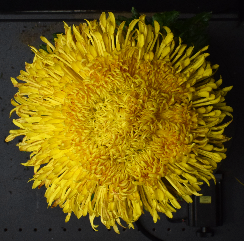 ‘Xiaonianjinmei’ |
| 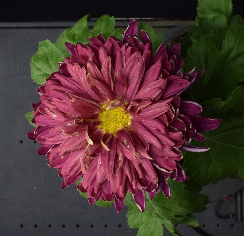 ‘Jinsihe’ | 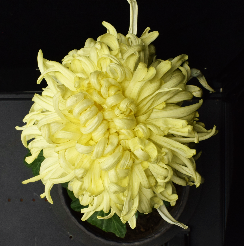 ‘Nenhuanghe’ | 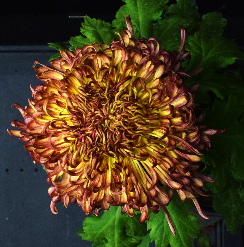 ‘Xingshitu’ | 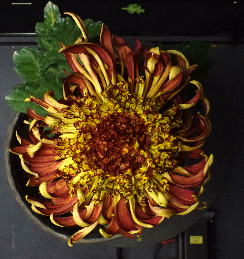 ‘Qiujuwanhong’ |
| 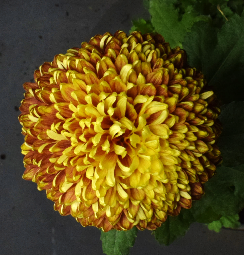 ‘Nijinqiehua’ | 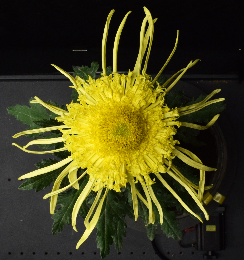 ‘Yuemingxingxi’ | 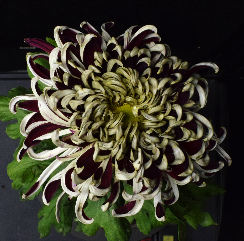 ‘Huige’ | 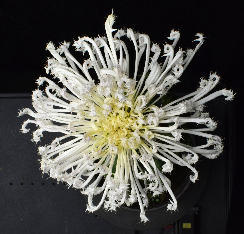 ‘Baimaoci’ |
| 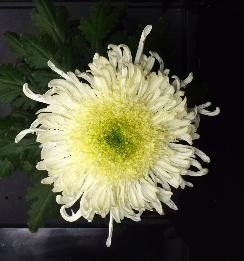 ‘Yinpantuogui’ | 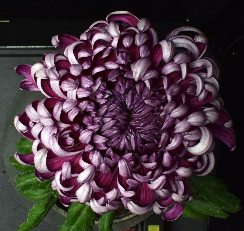 ‘Zichendian’ | 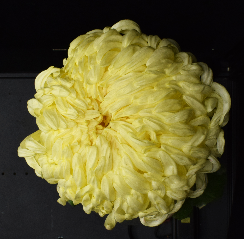 ‘Jinfomian’ | 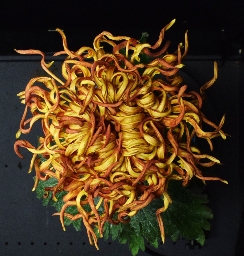 ‘Fenghuangyi’ |
| 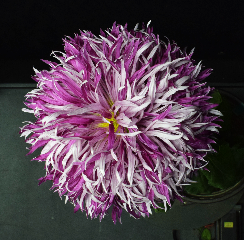 ‘Taobaochunfeng’ | 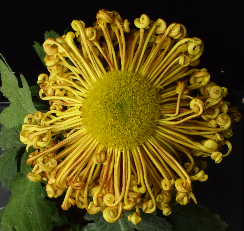 ‘Shibafenghuan’ | 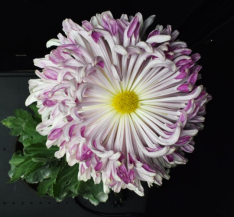 ‘Tangyufenfeiwu’ | 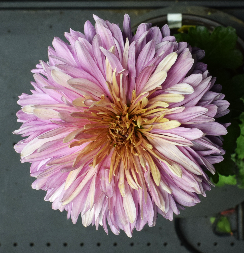 ‘Taohuahong’ |
| 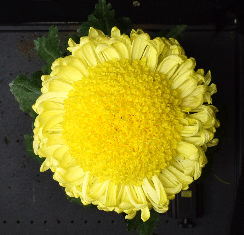 ‘Yuezhonggui’ | 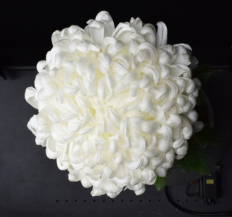 ‘Xuetao’ | 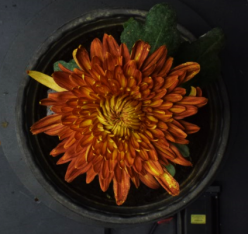 ‘Hongyun’ | 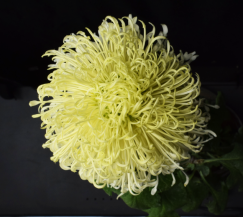 ‘Yuxianyinzhen’ |
| 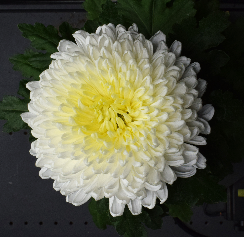 ‘Bingqingyujie’ | 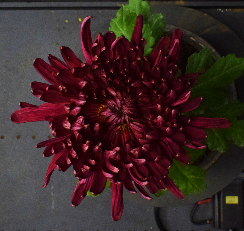 ‘Mojianrong’ | 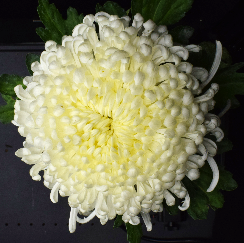 ‘Tangyuqinglian’ | 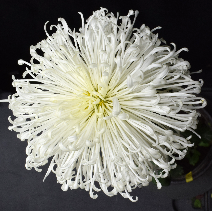 ‘Baisongzhen’ |
| 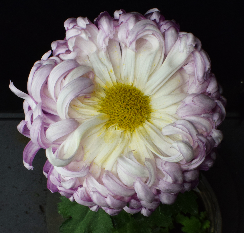 ‘Runmianhanqing’ | 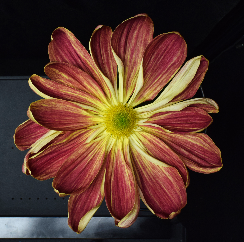 ‘Hongshiba’ | 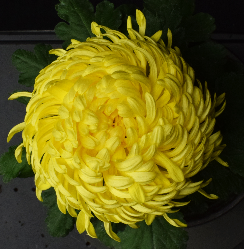 ‘Huangjinzhuangshi’ | 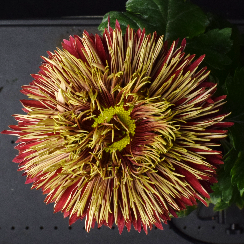 ‘Dianjiangchun’ |
| 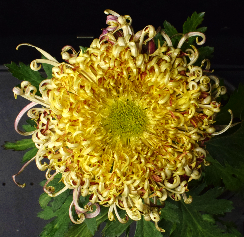 ‘Damofeiying’ | 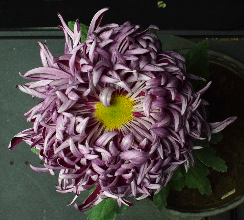 ‘Molanshuang’ | 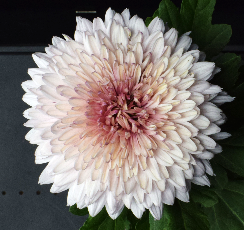 ‘Annigongzhu’ | 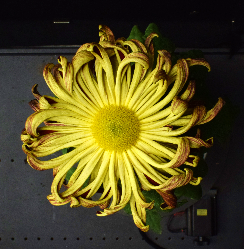  ‘Junqi’ |
| 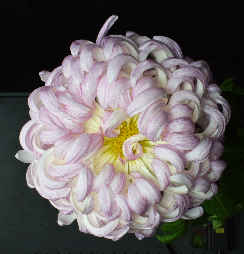 ‘Wenbixishang’ | 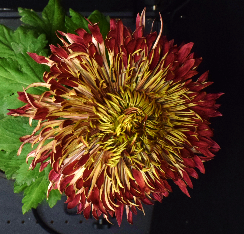 ‘Zhubidianchun’ | 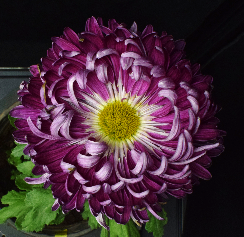 ‘Fenzichiguan’ | 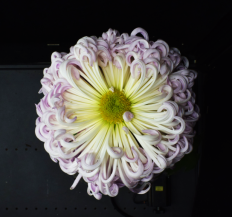 ‘Tangyuqiuyun’ |
| 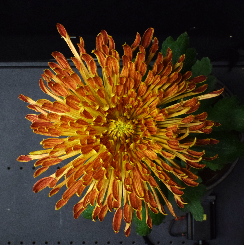 ‘Zhushahongshuang’ | 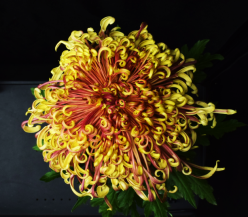 ‘Fenghuangzhenyu’ | 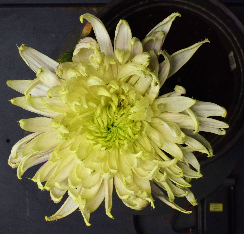 ‘Maguxianrui’ | 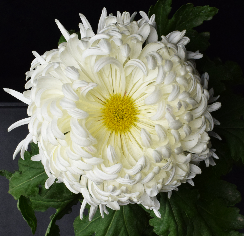 ‘Zilangfengguang’ |
| 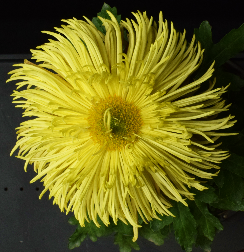 ‘Riluojinshan’ | 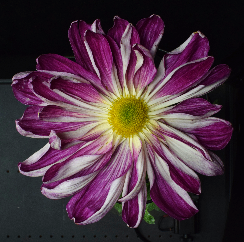 ‘Fenshiba’ | 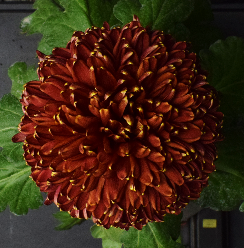 ‘Fengxian’ | 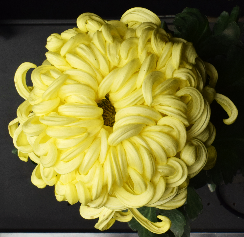 ‘Tangyujinqiu’ |
| 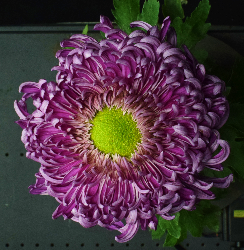 ‘Qingtangfenxie’ | 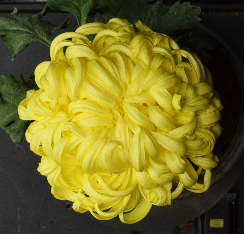 ‘Jinshitou’ | 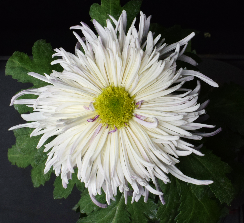 ‘Oufentuogui’ | 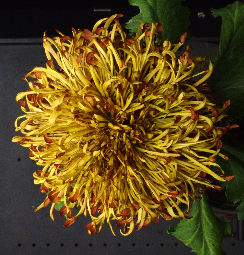 ‘Nijinshizi’ |
| 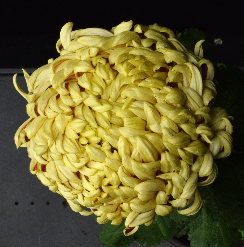 ‘Nijinbao’ | 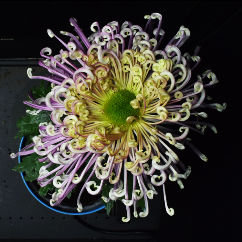 ‘Gushuiliuxia’ | 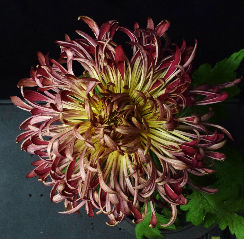 ‘Jinxiangyun’ | 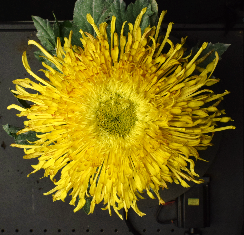 ‘Jintanzhua’ |
| 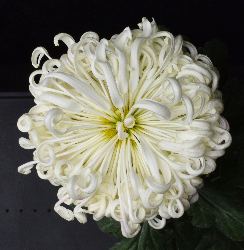 ‘Fengguanxiapei’ | 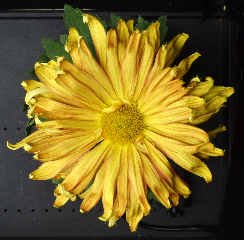 ‘Jinhongmaoci’ | 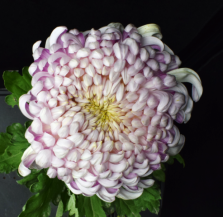 ‘Tangyuqiushi’ | 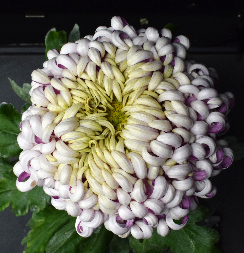 ‘Zilongwoxue’ |
| ‘Yanzhidianxue’ | ‘Gushuichunxia’ | ‘Zuiwuyangfei’ |  |

**Figure S1** Device images of 103 chrysanthemum cultivars (Top-view image)

Note: The images have been cropped.

LED light source

Camera 3

The up-down moving device

Camera 1

Camera 2

Control box

Computer monitor

Horizontal rotation platform

**Figure S2** Schematic diagram of the chrysanthemum image acquisition device

**Figure S3** Automatic chrysanthemum image acquisition device
